# Supplementary material for: Metagenomic and Antibiotic Resistance Analysis of the Gut Microbiota in Larus relictus and Anatidae Species Inhabiting the Honghaizi Wetland of Ordos, Inner Mongolia, from 2021 to 2023
Source: Microorganisms. 2024 May 13;12(5):978. doi: 10.3390/microorganisms12050978 (PMC11123678; doi:10.3390/microorganisms12050978)
Supplement: Supplementary file 1 [file microorganisms-12-00978-s001.zip › Supplementary Materials Table S1.pdf]

Supplementary Materials

Table S1. Statistics of antibiotic susceptibility testing of *Escherichia coli.*

Patterns of Drug Resistance	Number of Strains (strains)
/	106
TET	4
ATM-PIP-TZP	1
AMP-CHL-TET	1
CIP-LVX-MXF-TET	1
CZO-CTX-FEP-AMP-PIP	1
CZO-CTX-FEP-ATM-AMP-PIP	1
AMP-PIP-STX-CIP-LVX-MXF-TET	2

Note：TET, tetracycline; ATM, Aztreonam; PIP, Piperacillin; TZP, Piperacillin-Tazobactam; AMP, Ampicillin; CHL, Chloramphenicol; CIP, Ciprofloxacin; LVX, Levofloxacin; MXF, Moxifloxacin; CZO, Cefazolin; CTX, Cefotaxime; FEP, Cefepime; /, No resistant phenotype.
