# Supplementary material for: Metagenomic and Antibiotic Resistance Analysis of the Gut Microbiota in Larus relictus and Anatidae Species Inhabiting the Honghaizi Wetland of Ordos, Inner Mongolia, from 2021 to 2023
Source: Microorganisms. 2024 May 13;12(5):978. doi: 10.3390/microorganisms12050978 (PMC11123678; doi:10.3390/microorganisms12050978)
Supplement: Supplementary file 1 [file microorganisms-12-00978-s001.zip › Supplementary Materials Table S2.pdf]

Supplementary Materials

Table S2. Biofilm production of *E. coli* isolates in microtiter plates.

**Biofilm Production**	***E. coli*** **(*n* = 117)**
non-adherent	74.4%
weakly adherent	5.98%
moderately adherent	18.77%
strongly adherent	0.85%
