# Supplementary material for: Metagenomic and Antibiotic Resistance Analysis of the Gut Microbiota in Larus relictus and Anatidae Species Inhabiting the Honghaizi Wetland of Ordos, Inner Mongolia, from 2021 to 2023
Source: Microorganisms. 2024 May 13;12(5):978. doi: 10.3390/microorganisms12050978 (PMC11123678; doi:10.3390/microorganisms12050978)
Supplement: Supplementary file 1 [file microorganisms-12-00978-s001.zip › Supplementary Materials Table S3.pdf]

Supplementary Materials

Table S3. ***E. coli*** **16S rDNA primer sequences.**

Name of Primer	Primer Sequences (5’-3’)	Sequence Fragment Size	Reference
E. coli **16S rDNA**	**F：TGTGGGAACGGCGAGTCGGAATAC**<br>**R：GGGCGCAGGGGATGAAACTCAAC**	**1467 bp**	[1]

Note: F, forward; R, reverse.

REFERENCES

1. Shahi SK, Singh VK, Kumar A. Detection of Escherichia coli and Associated β-Lactamases Genes from Diabetic Foot Ulcers by Multiplex PCR and Molecular Modeling and Docking of SHV-1, TEM-1, and OXA-1 β-Lactamases with Clindamycin and Piperacillin-Tazobactam. *Plos One* 2013; **8**: e68234.
